# Zebrafish Egg Infection Model for Studying *Candida albicans* Adhesion Factors

**DOI:** 10.1371/journal.pone.0143048

**Published:** 2015-11-16

**Authors:** Yin-Zhi Chen, Yun-Liang Yang, Wen-Li Chu, May-Su You, Hsiu-Jung Lo

**Affiliations:** 1 National Institute of Infectious Diseases and Vaccinology, National Health Research Institutes, Miaoli, Taiwan; 2 Department of Biological Science and Technology, National Chiao Tung University, Hsinchu, Taiwan; 3 Institute of Molecular Medicine and Bioengineering, National Chiao Tung University, Hsinchu, Taiwan; 4 Institute of Molecular and Genomic Medicine, National Health Research Institutes, Miaoli, Taiwan; 5 School of Dentistry, China Medical University, Taichung, Taiwan; Louisiana State University, UNITED STATES

## Abstract

Disseminated candidiasis is associated with 30–40% mortality in severely immunocompromised patients. Among the causal agents, *Candida albicans* is the dominant one. Various animal models have been developed for investigating gene functions in *C*. *albicans*. Zebrafish injection models have increasingly been applied in elucidating *C*. *albicans* pathogenesis because of the conserved immunity, prolific fecundity of the zebrafish and the low costs of care systems. In this study, we established a simple, noninvasive zebrafish egg bath infection model, defined its optimal conditions, and evaluated the model with various *C*. *albicans* mutant strains. The deletion of *SAP6* did not have significant effect on the virulence. By contrast, the deletion of *BCR1*, *CPH1*, *EFG1*, or *TEC1* significantly reduced the virulence under current conditions. Furthermore, all embryos survived when co-incubated with *bcr1/bcr1*, *cph1/cph1 efg1/efg1*, *efg1/efg1*, or *tec1/tec1* mutant cells. The results indicated that our novel zebrafish model is time-saving and cost effective.

## Introduction

The prevalence of invasive fungal infections has increased substantially because the size of populations at risk have increased [1, 2]. The use of antifungal drugs and the incidence of drug resistance have increased concurrently [3, 4]. The limited variety of antifungal drugs and the emergence of drug resistance emphasize the importance of novel antifungal agents. Disseminated candidiasis is associated with 30–40% mortality in severely immunocompromised patients and *Candida albicans* is one of frequently isolated fungal pathogenic species in humans [5–7].

Mouse models are predominantly used for studying *C*. *albicans* pathogenesis, but are limited by difficulties in performing large-scale studies, high costs, and being time-consuming. To overcome these limitations, investigators have developed several invertebrate models, such as fruit flies (*Drosophila melanogaster*), nematodes (*Caenorhabditis elegans*), and larvae of wax moths (*Galleria mellonella*) [8–13] models. They feature conserved innate immunity and inexpensive care systems and enable experiments to be performed on a large scale [14].

Zebrafish (*Danio rerio*) are used in biomedical research because manual experimentation and drug administration are easy and the fish exhibits prolific fecundity. In addition, the optical transparency of zebrafish embryos enables real-time visualization of host-pathogen interactions. Chao et al. demonstrated that *C*. *albicans* could colonize and invade zebrafish at multiple anatomical sites, and caused mortality after being injected into the peritoneal cavities of adult fish or the yolks of embryos [15]. Brothers et al. subsequently observed that bath infections of zebrafish were not associated with mortality or fungal invasion, and developed a hindbrain ventricle infection model by using larvae [16]. The disadvantages of an injection model include unavoidable damage caused by injection, stringent technical requirements, and the time-consuming procedures. Gratacap et al. developed a noninvasive model of mucosal candidiasis of the swimbladder, natural infection site for *C*. *albicans*. [17]. However, this model did not cause mortality, which is a limitation for investigating virulence even though it is an appropriate model for studying immunity.

In this study, we established a zebrafish egg bath infection model as a simple noninvasive model for investigating *C*. *albicans* pathogenesis. *Candida albicans* switches between the unicellular yeast and the filamentous forms to adapt to various conditions. This switch is induced by many environmental cues. The induction by serum or by macrophages may be the most critical ones for the pathogenicity of *C*. *albicans* [18, 19]. The *cph1/cph1 efg1/efg1* double mutant fails to form filaments *in vitro* and does not cause lethal infections in a mouse model [20, 21]. These findings suggest that *C*. *albicans* strains possessing the ability to switch between the yeast and filament forms are those capable of penetrating vital organs and proliferating sufficiently to cause lethal infections. Even though the null mutation of *EFG1* but not *CPH1* affected the virulence of *C*. *albicans* in a mouse systemic infection model [21], the *cph1/cph1* mutant cells exhibited decreased virulence in a fly infection model [22]. In addition to *cph1/cph1*, *efg1/efg1*, *cph1/cph1 efg1/efg1* mutants, several other mutants were applied in the present study to evaluate this newly established model. Sap6p is a member of the secreted aspartyl protease family [23] and is highly up-regulated in biofilms [24]. However, the deletion of *SAP6* exerted no significant effects on germ tube and hyphal formation as well as on virulence in a mouse model [25]. Hyphal formation was defective in *tec1/tec1* mutant cells in liquid media but not solid ones [21, 26]. *BCR1* deletion caused defects in adhesion and biofilm formation but not in hyphal growth [27–29]. Our results show that this model enables the observation of hyphal/biofilm formation on the chorion and identification of the genes involved in adhesion.

## Materials and Methods

### Strains and media

The *C*. *albicans* strains used were the SC5314 wild type (WT) [30]; JKC19, *cph1/cph1* [31]; HLC52, *efg1/efg1* [21]; HLC54, *cph1/cph1 efg1/efg1* [21]; CAY3672, *bcr1/bcr1* [32]; DSY346, *sap6/sap6* [33]; CAY2504, *tec1/tec1* [32]; CAF2-dTomato [34] and OG1[15]. Yeast peptone dextrose (1% yeast extract, 2% peptone, and 2% dextrose), Roswell Park Memorial Institute 1640 medium (RPMI) (31800–022, GibcoBRL), and 10% fetal bovine serum (FBS) (GibcoBRL, US-628531) were prepared as described previously [35]. The compounds supplementing the media were obtained from Difco unless otherwise stated. *Candida albicans* cells were grown on YPD agar medium at 30°C for 1 day. Then, *C*. *albicans* were grown overnight with shaking in YPD broth at 30°C to stationary phase and the cell density of the inoculum of each strain was determined by OD_600_ and confirmed by plating.

### Zebrafish egg bath infection model

Wild-type zebrafish (*Danio rerio*), aged approximately 8–15 months were maintained in the zebrafish core facility at National Health Research Institutes (NHRI) (http://www.zebrafish-nthu-nhri.org/tzcf/) at 28°C in a 10-h dark 14-h light cycle. The fish were maintained according to maintenance and culturing procedures described previously [36]. Embryos were obtained from natural mating and staged according to the procedure described by Kimmel et al. [37]. One day post-fertilization, the embryos were sterilized using 0.028% chlorine bleach containing 0.0017% sodium hypochlorite to reduce the possibility of contamination. Data were derived from ≥ 2 repeated experiments unless otherwise stated.

The effect of 0.028% chlorine bleach to chorion structure was determined by adding fluorescein (CAS No. 2321-07-5) to a final concentration of 0.1 mg/mL after the bleach treatment. After one additional day of incubation, representative embryos were anesthetized in 0.2 mg/mL Tris-buffered tricaine methane sulfonate (Fluka A5040, China) and further immobilized in a mixture of 1% low-melting point agarose (Sigma A9414, St. Louis, MO, USA) in egg water. A Leica TCS SP5 II inverted microscope was used for confocal imaging. The green fluorescein was detected by optical filters for excitation/emission at 500 nm/550 nm. The distance between two slices was approximately 5 μm.

In general, after co-incubation, non-adhered *C*. *albicans* cells were removed from embryos by washing with egg water 3 times. Embryos incubated in 1 mL of egg water in 24-well plates at 30°C were imaged daily under an inverted microscope, with a beating heart used to indicate viability.

For the investigation of optimal conditions for conducting the egg infection model, embryos were placed in a 125 mL flask with 10 mL media containing 1 × 10^6^ or 1 × 10^7^ cells/mL of SC5314. The media used in the evaluations were egg water (0.03% sea salt), egg water containing 10% FBS (egg water/serum), RPMI, and RPMI containing 10% FBS (RPMI/serum). The embryos and *C*. *albicans* cells were co-incubated at 30°C for 1 h or 4 h with or without shaking at 80 or 180 repetitions per minute (rpm). After non-adhered *C*. *albicans* cells were removed, the embryos were incubated in egg water for additional 2 days. Since the laboratory standard wild-type SC5314 strain was used for identifying the conditions for conducting the infection model, we used approximately 10 embryos for each treatment.

To determine whether *C*. *albicans* hyphae were inside larvae, we co-incubated embryos with 1 × 10^6^ cells/mL of OG1 *C*. *albicans* cells in 6-well plates containing 4 mL of RPMI/serum with shaking at 80 rpm and 30°C for 4 h. After non-adhered *C*. *albicans* cells were removed, the embryos were incubated in egg water for additional 1 day. Representative embryos were anesthetized in 0.2 mg/mL Tris-buffered tricaine methane sulfonate and further immobilized in a mixture of 1% low-melting point agarose in egg water. The remaining embryos were used for the survival rate determination after 2 days additional incubation. A Leica TCS SP5 II inverted microscope was used for confocal imaging to determine the localization of *C*. *albicans* cells. The green OG1 *C*. *albicans* cells were detected by optical filters for excitation/emission at 500 nm/550 nm after 1 day additional incubation. The distance between two slices was approximately 5 μm.

To determine the lowest inoculum for conducting this model, embryos were co-incubated with 1 × 10^5^, 5 × 10^5^ or 1 × 10^6^ cells/mL of wild-type SC5314 or CAF2-dTomato *C*. *albicans* cells in 6-well plates containing 4 mL of medium with shaking at 80 rpm and 30°C for 4 h. Representative embryos co-incubated with CAF2-dTomato *C*. *albicans* cells were anesthetized in 0.2 mg/mL Tris-buffered tricaine methane sulfonate and further immobilized in a mixture of 1% low-melting point agarose in egg water. The remaining embryos were used for the survival rate determination after 2 days additional incubation. A Leica TCS SP5 II inverted microscope was used for confocal imaging to determine the localization of CAF2-dTomato *C*. *albicans* cells. The red CAF2-dTomato *C*. *albicans* cells were detected by optical filters for excitation/emission at 556 nm/656 nm after 1 day additional incubation. The distance between two slices was approximately 2 μm.

For evaluations of virulence, embryos were co-incubated with 5 × 10^5^ cells/mL of *C*. *albicans* in 6-well plates containing 4 mL of RPMI/serum and were shaken at 80 rpm and 30°C for 4 h. After non-adhered *C*. *albicans* cells were removed, the embryos were incubated in egg water at 30°C for additional 2 days. To make sure there were enough embryos to distinguish the level of virulence of various mutant strains, we used approximately 20 embryos for each treatment.

### Ethics Statement

The zebrafish protocol entitled “Evaluation of the functions of genes in *Candida* species using zebrafish models” (NHRI-IACUC-101071-A) was reviewed and approved by the Institutional Animal Care and Use Committee of the NHRI.

### Statistical analysis

The statistical significance of the differences in frequencies and proportions was determined by the log-rank test. A *p* value < 0.05 was considered significant.

## Results

### Optimal conditions for zebrafish egg bath infection

To determine the optimal conditions for zebrafish egg bath infection, we co-incubated wild-type *C*. *albicans* cells, SC5314, with 1-day post-fertilization embryos for various periods of time, at various shaking speeds, and in various media (Table 1). The embryos were imaged daily under an inverted microscope. We found that all embryos eventually hatched if they were not killed by *C*. *albicans* cells after an additional 2 days of incubation. Thus, the survival rates after an additional 2 days of incubation were discussed mainly in the present study.

**Table 1:** Conditions for the zebrafish egg bath infection model (mean of survival rate ± standard deviation).

	1-h co-incubation			4-h co-incubation		
	Shaking speed (rpm)						
Medium/inoculum	0	80	180	0	80	180
Additional 1 day of incubation						
EW/10^7^	100	100	100	100	100	100
EW+S/10^7^	90 ± 17.3*	57 ± 27.6	97 ± 5.8	77 ± 32.2	43 ± 51.3	67 ± 57.7
R/10^7^	96 ± 6.9	79 ± 20.1	93 ± 11.6	58 ± 45.2	7 ± 11.6	32 ± 33.1
R+S/10^7^	80 ± 34.6	73 ± 23.1	100	67 ± 49.3	33 ± 15.3	51 ± 39.1
R/10^6^	97 ± 5.8	92 ± 14.4	100	41 ± 31.0	14 ± 16.9	54 ± 40.8
R+S/10^6^	93 ± 11.6	97 ± 5.8	100	77 ± 40.4	29 ± 24.7	100
Additional 2 days of incubation							
EW/10^7^	92 ± 13.3	100	100	100	100	100
EW+S/10^7^	49 ± 19	11 ± 19.1	67 ± 57.7	62 ± 8.7	0	45 ± 50.7
R/10^7^	63 ± 55.1	17 ± 20.7	100	0	0	0
R+S/10^7^	60 ± 52.9	47 ± 21.6	83 ± 15.3	3 ± 5.8	0	29 ± 41.9
R/10^6^	57 ± 37.9	33 ± 57.7	100	0	0	37 ± 50.1
R+S/10^6^	53 ± 32.6	55 ± 50.7	72 ± 30.1	15 ± 13.8	0	91 ± 16.2

EW: egg water; R: RPMI; S: 10% FBS; 10^6^:1 × 10^6^ cells/mL;10^7^:1 × 10^7^ cells/mL

This data are from 3 repeat experiments. Approximately 30 embryos were tested for each treatment.

**Fig 1:**
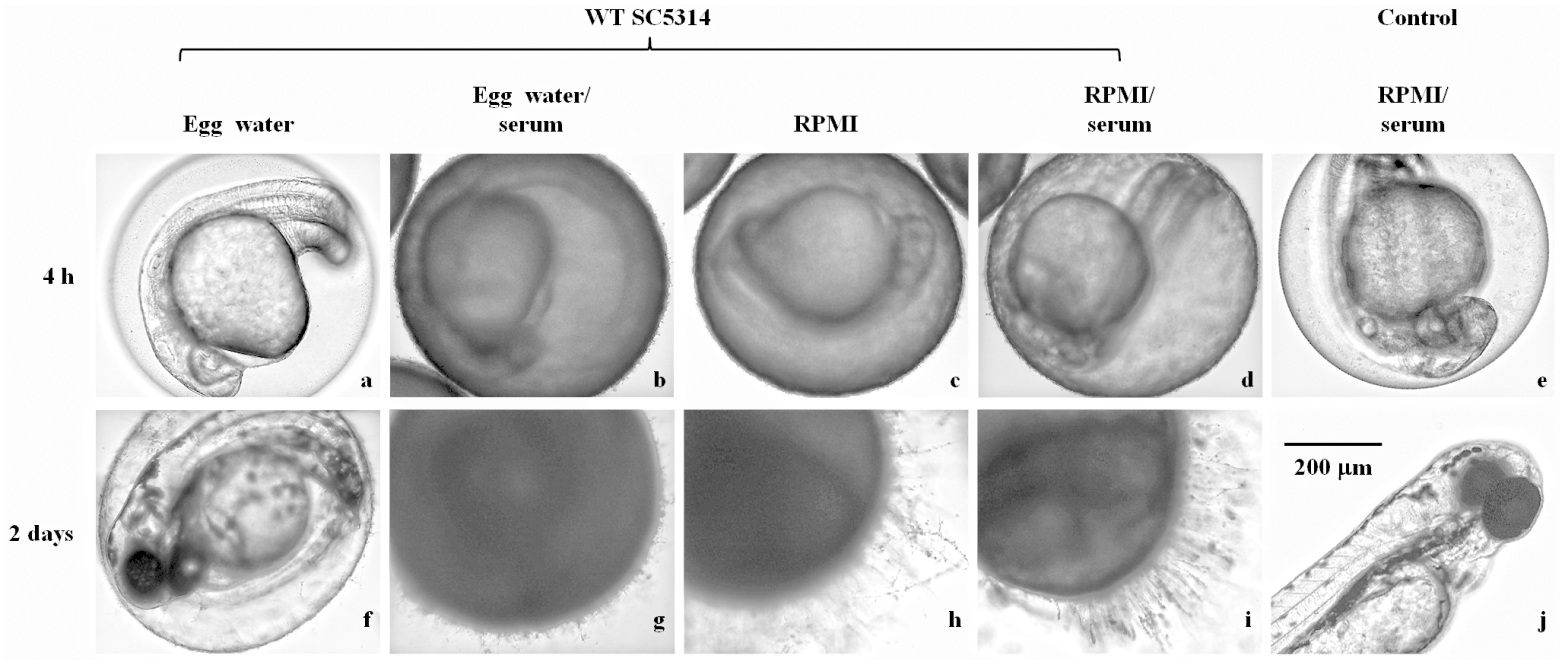
Zebrafish egg bath infection model in various media. Representative embryos were co-incubated with 1 × 10^6^ cells/mL of SC5314 (a-d, f-i) or without *C*. *albicans* (control, e, j) in egg water (a, f), egg water/serum (b, g), RPMI medium (c, h), RPMI/serum (d, i) with shaking at 80 rpm and 30°C for 4 h. The embryos were photographed immediately after non-adhered *C*. *albicans* cells were removed through washing (a-e) or after an additional 2 days of incubation (f-j). Scale bar = 200 μm. This data are from 3 repeat experiments. Approximately 30 embryos were tested for each treatment.

To determine whether bleach treatment affects the chorion membrane, like Dimethyl sulfoxide (DMSO) [38], we used fluorescein entrance as an indicator for chorion integrity. Even though bleach treatment affected chorion membranes and allowed fluorescein enter embryos, it did not kill embryos without the presence of *C*. *albicans* (Fig 1e and 1j). This result suggests that bleach-treated embryos can be used for establishing an animal model. All embryos in 11 of the 12 treatments survived when they were co-incubated with SC5314 in egg water alone irrespective of the shaking speed. SC5314 cells are arrested in egg water alone and adding serum to egg water allows the fungal cells to grow and form hyphae. After co-incubated embryos with SC5314 in egg water/serum for 4 h and applied shaking at 0, 80, and 180 rpm, we observed that the survival rates decreased to 62%, 0%, and 45%, respectively after an additional 2 days of incubation. Thus, addition of serum to egg water promotes the growth and/or adhesion capability of *C*. *albicans* cells.

The survival rates of the embryos co-incubated with SC5314 in RPMI or RPMI/serum without shaking for 1 h prior to an additional 2 days of incubation ranged from 53% to 63%. Under the same conditions but with longer co-incubation (4 h), the survival rates were from 0% to 15% (Table 1). Therefore, we observed higher mortality after 4 h co-incubation than after 1 h. With shaking to 80 rpm for 4 h, all embryos died in all treatments in RPMI or RPMI/serum (Table 1). When we increased the shaking speed to 180 rpm, the embryo survival rates increased from 0% to 29% (1 × 10^7^ inoculum in RPMI/serum), 37% (1 × 10^6^ inoculum in RPMI), and 91% (1 × 10^6^ inoculum in RPMI/serum) (Table 1). Hence, the sharking speed at 80 rpm appears to be suitable for conducting the experiment. Thus, the mixed embryos and *C*. *albicans* cells were shaken at 80 rpm for the initial infection in subsequent analyses. Since *C*. *albicans* cells formed hyphae and adhered on chorion in RPMI alone, adding serum did not affect mortality levels in RPMI.

**Fig 2:**
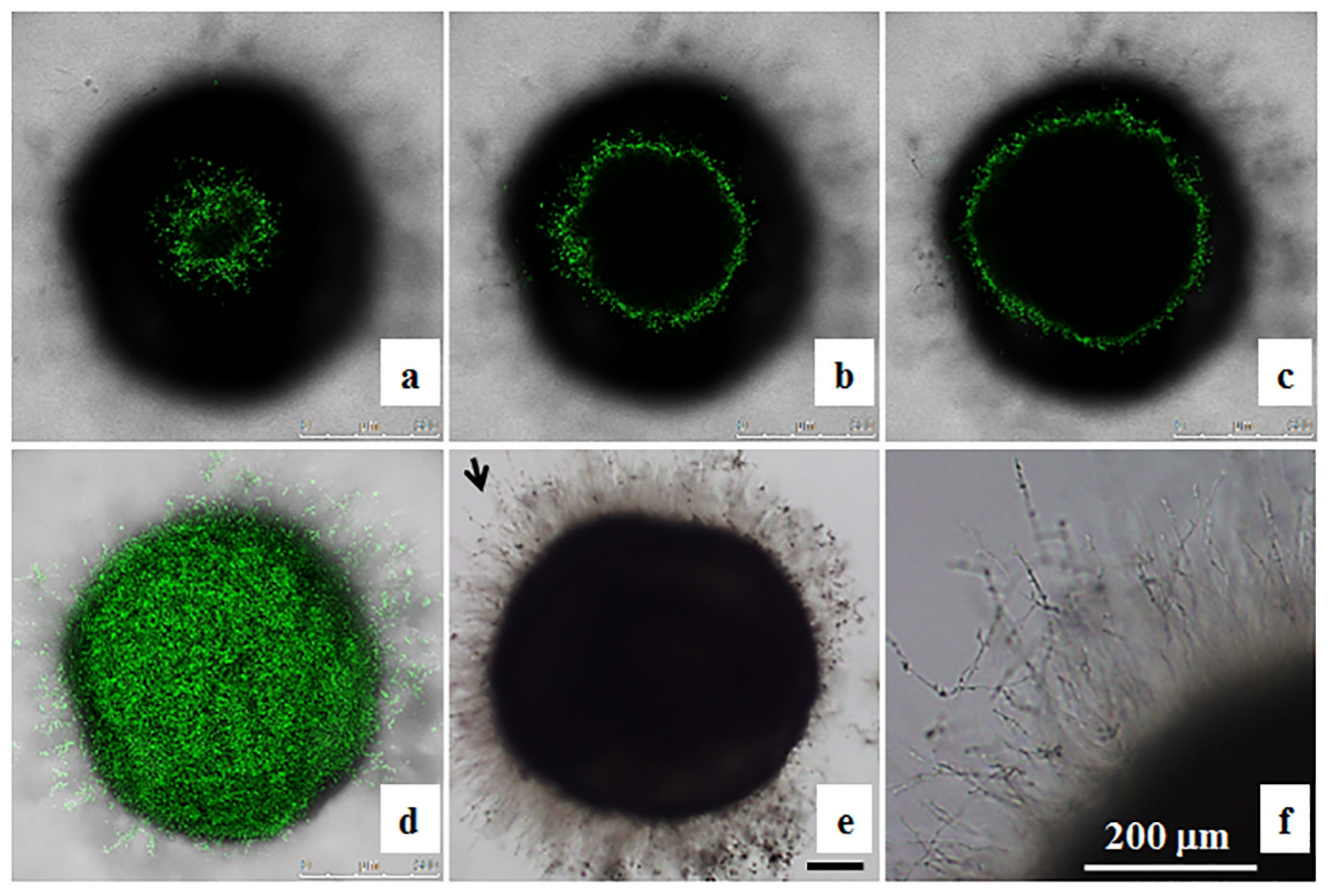
Localization of OG1 *Candida albicans* cells in zebrafish egg bath infection model. Embryos were co-incubated with 1 × 10^6^ cells/mL of OG1 *C*. *albicans*. The representative slices of confocal images (a-c) are shown. The distance between two slices was approximately 55 μm. The whole merged images are presented (d). The phase contrast photos showing *C*. *albicans* hyphae were taken by an inverted microscope (e, f). f is the enlargement of the arrow area in e. Scale bars = 200 μm.

The embryos co-incubated with *C*. *albicans* were examined under a microscope. We observed that abundant SC5314 cells were on the chorion of embryos immediately after 4 h co-incubation in egg water/serum (Fig 1b), RPMI (Fig 1c) or RPMI/serum (Fig 1d), but not in egg water alone (Fig 1a). After an additional 2 days of incubation in egg water, *C*. *albicans* cells co-incubated with embryos initially in RPMI (Fig 1h), or RPMI/serum (Fig 1i) formed more hyphae than those in egg water/serum (Fig 1g). Few *C*. *albicans* cells were detected on the chorion of embryos when co-incubated in egg water (Fig 1f), resulting in no mortality. Furthermore, we found that majority of *C*. *albicans* cells grew on the chorion and did not reach the embryo (Fig 2).

### Effects of gene deletion on *Candida albicans* virulence

**Fig 3:**
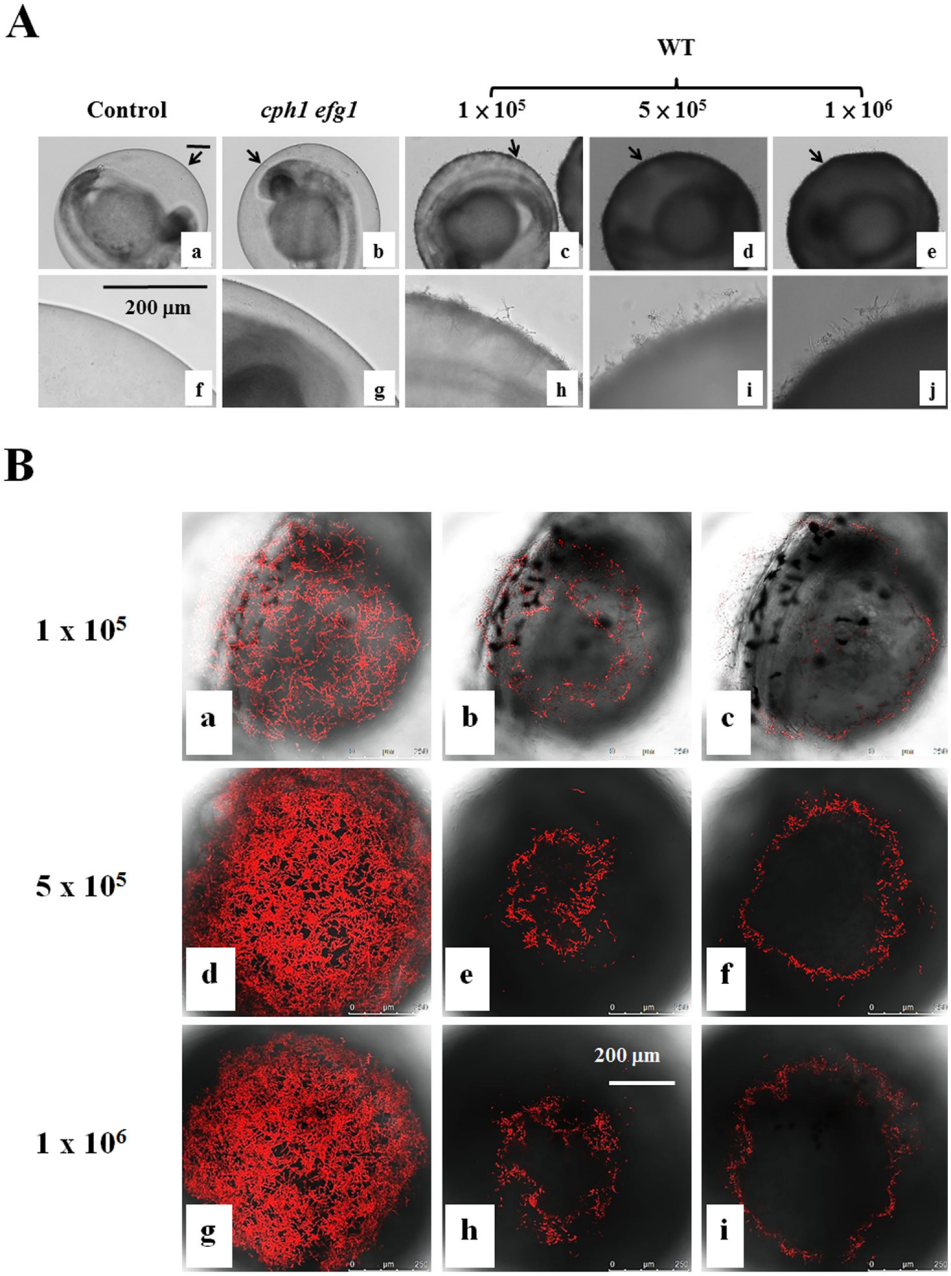
Zebrafish egg bath infection model with different inocula. (A) Embryos were co-incubated in the absence of *C*. *albicans* (a, f) or in the presence of 1 × 10^5^ (c, h), 5 × 10^5^ (d-i), or 1 × 10^6^ (e-j) cells/mL of wild-type SC531cells, 1 × 10^6^ (e-j) cells/mL of *cph1/cph1 efg1/efg1* mutant cells (b, g) for 4 h. f-j are the enlargement of the arrow areas in a-e. (B) Embryos were co-incubated with 1 × 10^5^ (a-c), 5 × 10^5^ (d-f), or 1 × 10^6^ (g-i) cells/mL of CAF2-dTomato *C*. *albicans*. The representative slices (b-c, e-f, h-i) are shown. The distance between two slices was approximately 16 μm. The whole merged images for 1 × 10^5^ (a), 5 × 10^5^ (d) or 1 × 10^6^ (g) cells/mL are presented. Scale bars = 200 μm.

The inocula of 1 × 10^6^ or 1 × 10^7^ cells/mL did not appear to have significantly different effects on the killing activity (Table 1). It is likely that the amount of *C*. *albicans* cells used has saturated the activity. Our preliminary data showed that abundant *C*. *albicans* cells were on the chorion and kill embryos in the inoculum equal to or greater than 5 × 10^5^ cells/mL, whereas, fewer *C*. *albicans* cells were detected on the chorion after 4 h co-incubation (Fig 3A) and a high proportion of embryos survived in the inoculum of 1 × 10^5^ cells/mL. Thus, the inoculum of 5 × 10^5^ cells/mL was chosen to conduct subsequent experiments. Like OG1 cells, we found that majority of CAF2-dTomato *C*. *albicans* cells (Fig 3B) grew on the chorion and did not reach the embryo.

**Fig 4:**
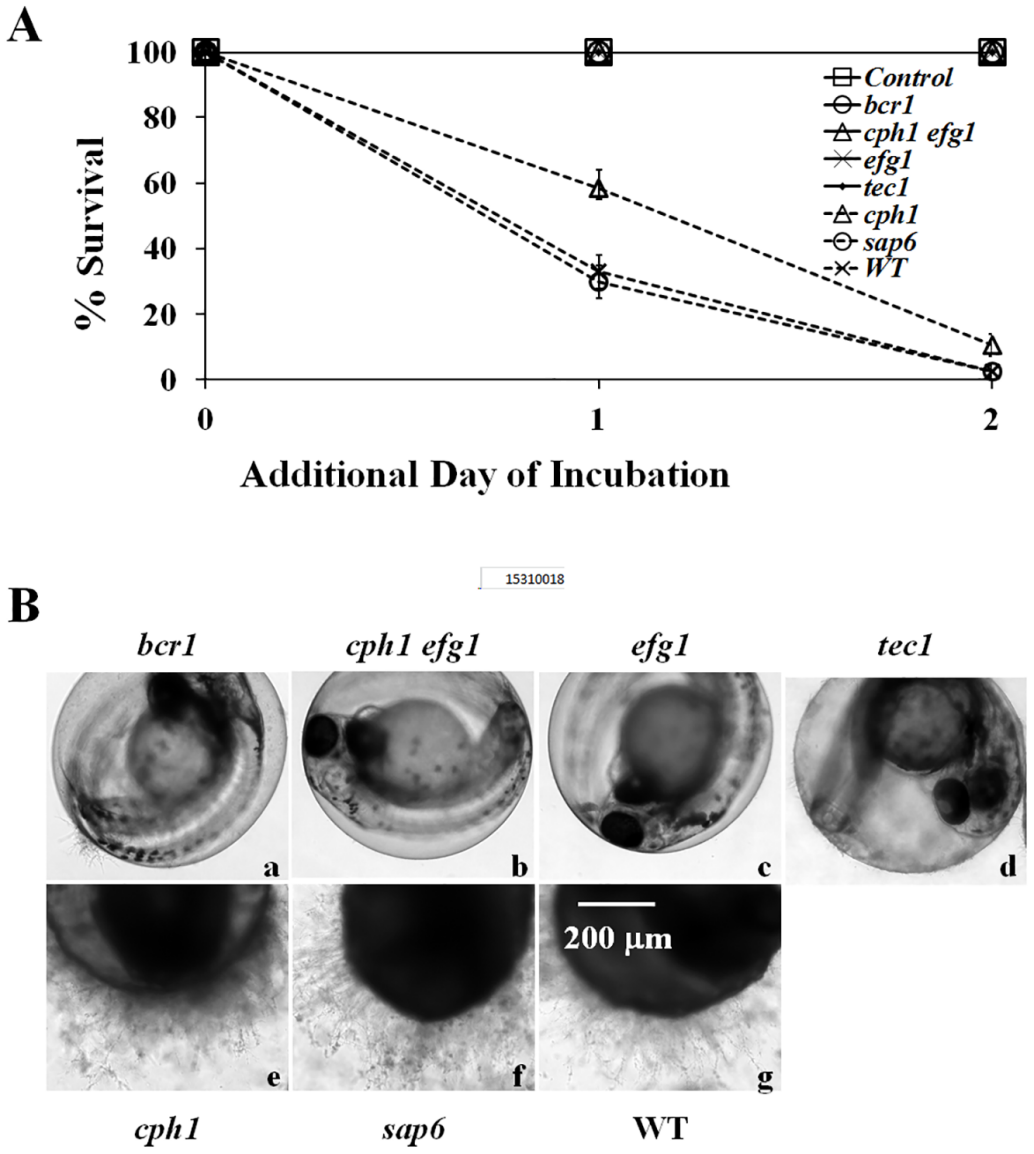
Virulence of *C*. *albicans* mutant strains in the infection model. (A) Survival rates of embryos. Embryos alone (Control) or embryos with 5 × 10^5^ cells/mL of *bcr1/bcr1*, *cph1/cph1 efg1/efg1*, *efg1/efg1*, *tec1/tec1*, *cph1/cph1*, *sap6/sap6*, or WT (SC5314) cells in RPMI/serum were incubated at 30°C for 4 h. Survival rates were determined after an additional 1 day and 2 days of incubation. (B) Representative embryos were co-incubated with (a) *bcr1/bcr1*, (b) *cph1/cph1 efg1/efg1*, (c) *efg1/efg1*, (d) *tec1/tec1*, (e) *cph1/cph1*, (f) *sap6/sap6*, or (g) WT (SC5314) cells, and photographed after an additional 2 days of incubation. Scale bar = 200 μm. The data are from 4 repeat experiments. Approximately 70 embryos were tested for each strain.

To evaluate our infection model, we examined the virulence of several *C*. *albicans* strains. Embryos co-incubated with various mutant strains in RPMI/serum instead of RPMI to mimic the bloodstream of human were shaken at 80 rpm at 30°C for 4 h. The results are summarized in Table 2 and Fig 4. Few embryos survived after co-incubated with the wild-type cells (Fig 4A, crosses with dot line) or *sap6/sap6* mutant cells (Fig 4A, circles with dot line). In contrast, the deletion of *BCR1*, *CPH1*, *EFG1*, and *TEC1* significantly reduced *C*. *albicans* virulence. More embryos survived when co-incubated with *cph1/cph1* mutant cells than those with wild-type SC5314 cells after an additional 1 day and 2 days of incubation. Thus, *cph1/cph1* mutant cells exhibited decreased level of virulence in this model (p = 0.0006). Furthermore, all embryos survived when co-incubated with the adhesion and biofilm deficient mutants, *bcr1/bcr1*, *cph1/cph1 efg1/efg1*, *efg1/efg1*, or *tec1/tec1* (p < 0.0001). Hence, the virulence levels of those strains in this model are Wild-type = *sap6/sap6* > *cph1/cph1* >> *bcr1/bcr1* = *cph1/cph1 efg1/efg1* = *efg1/efg1* = *tec1/tec1*.

**Table 2:** Mean of survival rates of embryos after co-incubated with various mutant strains.

	Control (n = 76)	*bcr1* (n = 71)	*cph1 efg1*(n = 72)	*efg1* (n = 73)	*cph1* (n = 77)		*sap6* (n = 81)		WT (n = 82)	
hours	Mean	Mean	Mean	Mean	Mean	SEM	Mean	SEM	Mean	SEM
0	100	100	100	100	100		100		100	
1 day	100	100	100	100	58.4	5.6	29.7	5.1	32.9	5.2
2 days	100	100	100	100	10.4	3.5	2.5	1.7	2.4	1.7

Like the wild-type SC5314 cells (Fig 4Bg), there were abundant *cph1/cph1* (Fig 4Be), and *sap6/sap6* (Fig 4Bf) hyphal cells on the chorion after an additional 2 days of incubation. Few or no *bcr1/bcr1* (Fig 4Ba), *cph1/cph1 efg1/efg1* (Fig 4Bb), *efg1/efg1* (Fig 4Bc), or *tec1/tec1* (Fig 4Bd) mutant cells were observed on the chorion. These mutant cells were not associated with mortality.

## Discussion

In this study, we identified the conditions (bleached treated 1 day old embryos co-incubated with 5 × 10^5^ cell/mL *C*. *albicans* at stationary phase in 4 mL RPMI or RPMI/serum at 80 rpm at 30°C for 4 h) for conducting zebrafish egg bath infection model. We also evaluated this model with various strains known to be defective in virulence in other models. To mimic the bloodstream of human, RPMI/serum was used in the present study. We observed an appropriate shaking speed not only mimic the condition of blood flow but also enable embryos to mix evenly with the *C*. *albicans* cells, as suggested previously [17]. This novel zebrafish model is time-saving and cost effective.

To establish an infection, pathogenic cells adhere to the surfaces of host epidermal or endothelial cells, invade host cells, evade the immune system, survive and propagate in the host environment, and then spread to new tissues [39]. However, whether adhesion and hyphal formation are essential for the lethality of *C*. *albicans* cells remains unclear. Our observation that *bcr1/bcr1* mutant cells were not lethal to the embryos suggests that the adhesion and biofilm formation capabilities during the co-incubation period are critical. The mouse systemic infection model is commonly used for investigating the functions of interested genes related to the virulence of *C*. *albicans*. However, when *C*. *albicans* cells are injected directly into the mouse tail vein, some of the genes involved in adhesion may not be detected. Our zebrafish egg bath infection model provides an alternative model to identify virulence genes, particularly those involved in adhesion. Since *C*. *albicans* hyphal cells did not reach the embryo, the potential cause(s) for the death of the embryos, including failure of transporting toxicities, either secreted by *C*. *albicans* or generated by embryos, and/or lack of oxygen are under investigation.

The morphological transition between yeasts and hyphae by *C*. *albicans*, generally considered essential for full virulence of this fungus [40], is induced by growth at 37°C and other stimuli, including serum, in vitro. Interestingly, we found that *C*. *albicans* can form hyphae at lower temperature, consistent with previous report [16]. Furthermore, we observed that *C*. *albicans* adhering to the chorion of embryos formed long hyphae/biofilms (Fig 4Be–4Bg) in egg water at 30°C, indicating that additional host-related factors from embryos are crucial for superseding the need for increased temperature and other stimuli, such as serum. Compared to other in vitro systems, such as polystyrene microplates, mammalian cell lines, and reconstituted human epithelium [41–43], the zebrafish egg bath model can be applied to identify the receptors on the chorion of the embryos which can be beneficial for future study of *C*. *albicans*-host interaction.
